# *Coxiella burnetii* in ticks, livestock, pets and wildlife: A mini-review

**DOI:** 10.3389/fvets.2022.1068129

**Published:** 2022-11-11

**Authors:** Seyma S. Celina, Jirí Cerný

**Affiliations:** Center for Infectious Animal Diseases, Faculty of Tropical AgriSciences, Czech University of Life Sciences Prague, Prague, Czechia

**Keywords:** *Coxiella burnetii*, Q fever, coxiellosis, ticks, livestock, wildlife

## Abstract

*Coxiella burnetii* is a zoonotic bacterium with an obligatory intracellular lifestyle and has a worldwide distribution. *Coxiella burnetii* is the causative agent of Q fever in humans and coxiellosis in animals. Since its discovery in 1935, it has been shown to infect a wide range of animal species including mammals, birds, reptiles, and arthropods. *Coxiella burnetii* infection is of public and veterinary health and economic concern due to its potential for rapid spread and highly infectious nature. Livestock are the primary source of *C. burnetii* infection in most Q fever outbreaks which occurs mainly through inhalation of contaminated particles. Aside from livestock, many cases of Q fever linked to exposure to wildlife. Changes in the dynamics of human-wildlife interactions may lead to an increased potential risk of interspecies transmission and contribute to the emergence/re-emergence of Q fever. Although *C. burnetii* transmission is mainly airborne, ticks may act as vectors and play an important role in the natural cycle of transmission of coxiellosis among wild vertebrates and livestock. In this review, we aim to compile available information on vectors, domestic, and wild hosts of *C. burnetii*, and to highlight their potential role as bacterial reservoirs in the transmission of *C. burnetii*.

## Introduction

*Coxiella burnetii*, a member of the *Coxiellaceae* family and the aetiologic agent of Q fever disease in humans and the epizootic disease coxiellosis in animals, is an obligate intracellular gram-negative bacterium. *Coxiella burnetii* infection occurs in a wide variety of animals such as mammals, birds, reptiles, and arthropods (1). Due to its widespread availability, environmental stability, and low infective dose, *C. burnetii* is reported as an emerging pathogen and classified as a potential bioterror agent (2).

**Figure 1:**
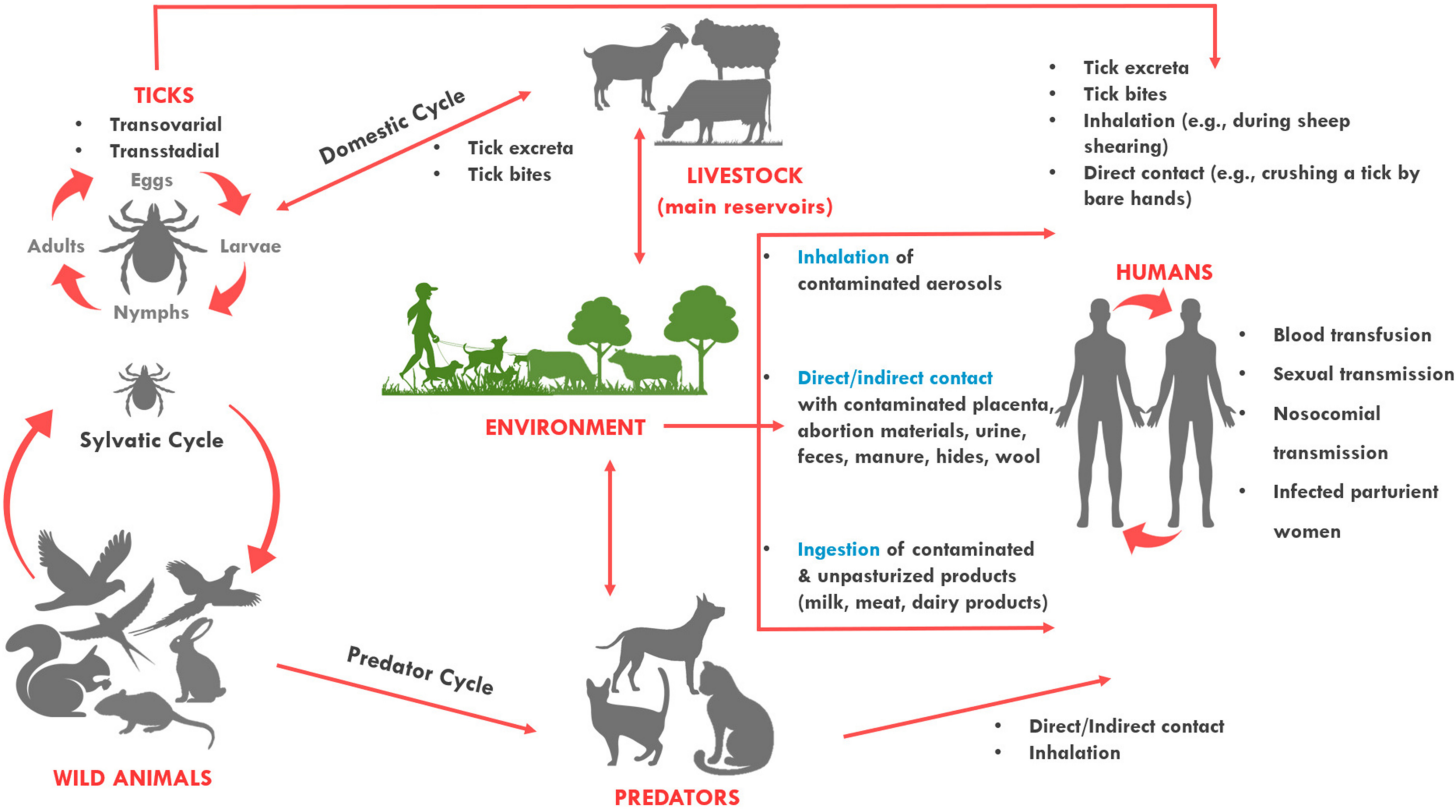
Transmission routes of *Coxiella burnetii*. The bacterium is most often transmitted to humans during parturition of animals. It can be transmitted to humans when they directly come into contact with or inhale contaminated dust from infected animals. It can also be transmitted from a tick bite or by ingesting contaminated/unpasteurized milk and dairy products. Among humans, the bacterium can be transmitted through blood transfusion, sexual, nosocomial and vertical transmission. Sheep, cattle, and goats are the most common reservoirs of the organism for human infection, but infected cats and less commonly dogs can also transmit the infection to humans. Ticks also harbor the organism and are thought to maintain transmission to wildlife species. Dogs and cats, and humans can also become infected when they contact (or ingest) wildlife species.

*Coxiella burnetii* has a wide and diverse host range. The pathogen primarily affects sheep, goats, and cattle which are considered the primary reservoirs of the pathogen and the primary source of human outbreaks (3, 4). *Coxiella burnetii* has a worldwide geographical distribution, apart from Antarctica and New Zealand (5, 6). People get infected through inhalation of bacteria contaminated aerosols expelled by infected animal feces, urine, milk, and birth products, while alternative routes of the infection such as sexual, oral, or congenital are uncommon (Figure 1) (7, 8). As the infective dose through inhalation is <10 bacterial cells, exposure to infected animals and their products poses a significant risk for acquiring the pathogen, particularly for farmers and veterinarians (9–11).

Apart from domestic ruminants, a diverse range of other domestic animals (e.g., dog, cat, rabbit, pig, horse, camel, buffalo, rodent, birds) have been reported to be infected with *C. burnetii*. Additionally, these animals have been also documented to spread the pathogen to humans without manifesting clinical signs of coxiellosis (1). Furthermore, more than a 100 different wild mammal species, which can act as reservoirs for both humans and domestic livestock, have been shown to harbor *C. burnetii* (12).

In this review, we provide compiled available information on vectors, domestic, and wild hosts of *C. burnetii*, and highlight their potential role as bacterial reservoirs in the transmission of *C. burnetii*. This review provides a short synopsis of much broader topics that have been covered in depth by recent publications (5, 12–17) for those who are interested in more details on *C. burnetii* in animals.

## *Coxiella burnetii* in animals

### *Coxiella burnetii* in ticks

Q fever was first recognized in 1,935 among abattoir workers in Australia by pathologist Derrick (18). Two years later, the pathogenic agent was first isolated from *Dermacentor andersonii* (Nine Mile isolate) (19) and *Haemaphysalis humerosa* (20) in the US. Since then, ticks have been discussed as possible vectors for transmission of the bacterium (21).

The role of ticks in Q fever epidemiology is being disputed due to the rare detection of *C. burnetii* in ticks. Also, difficulties in distinguishing *C. burnetii* from *Coxiella*-like endosymbionts (CLEs) of ticks, which are non-infectious for the vertebrate hosts but necessary for tick fitness, is problematic (13, 22). CLEs have a strong genetic similarity to *C. burnetii* therefore, routine PCR detection usually cross-react and its results may lead to misinterpretation of real prevalence of *C. burnetii* in ticks (23, 24). Moreover, in contrast to majority of vector-borne diseases, ticks are not essential as vector in transmission of *C. burnetii* (25). However, several studies have identified ticks as a potential risk for coxiellosis in livestock and other domestic animals (26–30). On the other hand, ticks may play a significant role as reservoirs of *C. burnetii* in wild animals (31).

*Coxiella burnetii* was isolated from over 40 species of hard ticks and 14 soft tick species collected from vegetation, and domestic and wild animals (25, 32). Ticks can get infected with *C. burnetii* either through a blood meal from an infected animal at all stages of their development or transovarially. However, under experimental conditions, not all tick species were capable of being infected or being able to transmit the pathogen to experimental animals or to their offspring (25). Seven species of hard and soft ticks, including *D. andersoni, Ha. humerosa, Hyalomma aegyptium, Hy. asiaticum, Ixodes holocyclus, Ornithodoros hermsi*, and *Or. moubata* have yet to be demonstrated in experiments to be competent vectors of *C. burnetii* (13). In ticks, *C. burnetii* has been detected in several tick tissues, including midgut, hemolymph, Malpighian tubules, salivary glands, and ovaries (33). Ticks have also been shown to excrete considerable amount of infectious feces (up to 10^10^ organisms per gram of feces) (34). This finding emphasizes the potential risk of tick-borne infection posed by tick excreta, through inhalation (e.g., among shearers), direct contact (e.g., using bare hands to crush a tick), or tick bites (13, 31, 35).

Nevertheless, from the perspective of public health, the epidemiological importance of *C. burnetii* tick-borne transmission is lower compared to airborne transmission (13). However, inhaling tick excreta can be a significant source of infection. Additionally, ticks may play a meaningful role in the natural cycle of transmission of coxiellosis among wild-living animals and livestock (31). By crossing these species barriers, *C. burnetii* may increase its diversity of virulence and resistance factors (13).

Furthermore, several studies showed variable *C. burnetii* prevalence in ticks collected from vegetation. Although the prevalence of *C. burnetii* in ticks sampled from animals (6.6%) is higher than in ticks collected from vegetation (2.8%) (36), the *C. burnetii* prevalence in ticks collected from vegetation varies geographically. For instance, *C. burnetii* could not detected in 1891 *I. ricinus* collected in the Netherlands (37), or 887 *I. ricinus* collected in Sweden (38). However, *C. burnetii* was detected in *I. ricinus, D. reticulatus, D. marginatus, Ha.concinna*, and *Ha. inermis* in Slovakia (39), in *I. ricinus, D. marginatus*, and *Ha.concinna* in Hungary (40), in *I. ricinus* in Germany (41), in *Ha. punctata* in Spain (42), and in *I. ricinus* in Austria (43). These results suggest that ticks may play an important role at least in the forest cycle of *C. burnetii* (31).

### *Coxiella burnetii* in livestock and domestic animals

As already mentioned, livestock is the most frequent reservoir of *C. burnetii* and frequent contacts with domestic ruminants are one of the most important risk factors for *C. burnetii* infection in humans. Therefore, the detection and control of infected herds is a critical issue in control of Q fever from the public health perspective (25). Massive excretion of *C. burnetii* into the environment occurs during parturition in infected females, with shedding a very large number of bacteria in birth products and in urine, feces, and milk. Shedding of *C. burnetii* may persist over several months in various body tissues and fluids such as vaginal mucus, feces, urine, and milk (44). According to experimental studies, *C. burnetii* shedding through vaginal discharge is more frequently observed in sheep, while milk is the most common route of pathogen shedding in cattle and goats (45–47).

In most Q fever outbreaks, infected ruminants are typically the primary source of infection (3, 4) and the number of cases is correlated to the local livestock population (48). Transmission of *C. burnetii* between ruminant hosts mainly occurs *via* the airborne route. However, other factors such as transboundary animal trade and transportation of animal source foods enhance movement of the pathogen across large distances (16, 49). Aside from livestock, pet animals, especially dogs and cats kept in a close contact with their owners, are known to be important reservoirs of *C. burnetii* during urban Q fever outbreaks. Many cases of human Q fever have been reported from infected dogs and cats (15, 50–57). Most of these reported cases of Q fever in humans were associated with exposure to parturient pets. Although the main origins of infection in pets remain poorly understood, it has been speculated that dogs and cats may be infected by tick bite, consumption of placenta or milk from infected livestock, consumption of raw meat, inhalation of aerosolized bacteria in the environment, and preying of contaminated animal species (16, 17).

*Coxiella burnetii* infection has been reported in other domestic mammals—though less frequently than livestock —, including horses, rabbits, pigs, camels, water buffalo, rats, and mice (11, 30, 35, 58–72). So far, serological evidence of *C. burnetii* infection in many horses have been reported (66, 73–75). However, the epidemiological importance of horses as *C. burnetii* reservoirs has not been adequately studied.

The role of European rabbits as a reservoir for *C. burnetii* was evaluated and rabbits were identified as a major source of coxiellosis in livestock and Q fever in humans in Europe, and it is possible that they have a similar role in Australia (68, 76).

The role of pigs in epidemiology of *C. burnetii* is relatively obscure. Their susceptibility to *C. burnetii* infection has been confirmed based on previous serological study (77), but there is a lack of evidence that pigs serve as reservoirs of *C. burnetii*. Until today, no *C. burnetii* transmission from pigs to humans has been documented. The study on evaluation of the prevalence and genotypes of *C. burnetii* in pigs from South Korea revealed low seropositivity among pigs (59).

Domestic water buffalo (*Bubalus bubalis*) is a globally important livestock species due to its high-quality milk, meat and leather. Many studies revealed that buffalo may have a significant impact on the epidemiology of Q fever and emphasizes the necessity for *C. burnetii* surveillance and control measures in buffalo (60, 65–67).

In camels, *C. burnetii* is among the most widespread zoonotic pathogens (61). Camel populations worldwide are estimated to be ~30 million, with dromedary camels constituting 95% of the population (78). Dromedary camels (*Camelus dromedarius*) play an important role as a high-quality protein source for people in semi-dry and arid zones of Africa (5). The most recent *C. burnetii* serological studies conducted in herds and farms in Africa, Arabian Peninsula, and Asia reported a very high prevalence of Q fever antibodies in the sera of camels (5, 79, 80).

The pathogenesis of Q fever in domestic animals is not fully understood. *Coxiella burnetii* infections in animals are frequently asymptomatic. In the acute phase, *C. burnetii* can be detected in the blood, lungs, spleen, and liver (17). When persistent shedding of bacterium occurs in feces and urine, the infection often becomes chronic. However, unlike humans, animals do not usually develop chronic endocarditis (17). Nonetheless, recent studies reported *C. burnetii* detection in inflamed cardiac valves in slaughtered cattle (81) as well as chronic endometritis in dairy cattle, resulting chronic subfertility (82). Chronic *C. burnetii* infection mostly affects female uterus and mammary glands. Therefore, the most significant clinical cases of *C. burnetii* infection is limited to pregnant animals and are commonly associated with abortions, stillbirths, the birth of small or weak offspring, and mastitis (17, 25).

In laboratory animals, *C. burnetii* inoculation of guinea pigs and mice leads to pneumonia, hepatitis and splenomegaly (83, 84). In addition, the importance of *C. burnetii* strain in determining the severity of pathological changes was reported (17). Splenomegaly in guinea pigs and mice is considered to be an indication of *C. burnetii* strain pathogenicity. Furthermore, the method of inoculation appears to impact pathogenesis. In mice, intranasal inoculation causes pneumonia, whereas intraperitoneal inoculation causes hepatosplenomegaly (84).

Little is known about the pathogenesis of *C. burnetii* infections in companion animals. In pet animals, especially dogs and cats, reproductive anomalies, including dystocia, stillbirths and perinatal mortality have been recorded. However, many studies reported short-lived bacterial shedding and indicated that bacterial shedding by companion animals is rare given the lack of *C. burnetii* DNA in samples from companion animals (85).

### *Coxiella burnetii* in wildlife

*Coxiella burnetii* occurs in many free-living and captive wildlife species worldwide that have been suspected to play a role in the epidemiology of Q fever (12, 86–92). González-Barrio and Ruiz-Fons discussed in details about how it is exceedingly difficult to identify *C. burnetii* infection in wildlife, particularly farmed wild species and free-roaming wildlife (12, 93, 94), and highlighted the importance of daily survey to easily detect reproductive disorders associated to *C. burnetii* in captive animals of zoological gardens (12).

Natural infections of *C. burnetii* have been reported in a large variety of wild species (Table 1). Starting with birds, *C. burnetii* infection has been documented in avian wildlife in addition to farm and pet birds (14, 98)—e.g., barn swallow (*Hirundo rustica*), eclectus parrot (*Eclectus roratus*), hen (*Gallus gallus domesticus*), turkey (*Meleagris* spp.), magpie (*Pica* spp.), pheasant (*Phasianus colchicus*), wood-pigeon (*Columba palumbus*), turtle dove (*Streptopelia turtur*), pigeon (*Columba livia*), house sparrow (*Passer domesticus*), Italian sparrow (*Passer italiae*), rook (*Corvus frugileus*), hooded crow (*Corvus cornix*), carrion crow (*Corvus corone*), raven (*Corvus corax*), Eurasian griffon vulture (*Gyps fulvus*), black kite (*Milvus migrans*), redstart (*Phoenicurus phoenicurus*), white wagtail (*Motacilla alba*), western yellow wagtail (*Motacilla flava*), common quail (*Coturnix coturnix*), Japanese quail (*Coturnix japonica*), black-headed gull (*Chroicocephalus ridibundus*), common gull (*Larus canus*), white-winged tern (*Chlidonias leucopterus*), common tern (*Sterna hirundo*), common starling (*Sturnus vulgaris*), wild ducks (*Anas* spp.), common blackbird (*Turdus merula*), fieldfare (*Turdus pilaris*), thrush nightingale (*Luscinia luscinia*), willow warbler (*Phylloscopus trochilus*), great white pelican (*Pelecanus onocrotalus*), Eurasian reed warbler (*Acrocephalus scirpaceus*), and wood sandpiper (*Tringa graleola*). Ebani and Mancianti reviewed *C. burnetii* infections in birds from 1952, when they were first documented, to the present, and supports the potential role of avian populations in the epidemiology of *C. burnetii* (14). However, it is yet unclear how *C. burnetii* spreads among avian wildlife and what factors affect the transmission of the pathogen, as information about prevalence rates in different geographic locations is scarce (14).

**Table 1:** List of animal species infected by *Coxiella burnetii*.

**Family**	**Common name**	**Scientific name**	**Country**	**Reference**
***Coxiella burnetii infection in birds***				
Accipitridae	Black kite	*Milvus migrans*	Spain	(95)
	Eurasian griffon vulture	*Gyps fulvus*	Spain	(95)
Acrocephalidae	Eurasian reed warbler	*Acrocephalus scirpaceus*	Bulgaria	(96)
Anatidae	Eurasian teal	*Anas crecca*	Italy	(97)
	Eurasian wigeon	*Anas penelope*	Italy	(97)
	Mallard	*Anas platyrhynchos*	Russia	(96)
			Japan	(98)
Columbidae	Pigeon	*Columba livia*	Bulgaria	(99)
			Czechoslovakia	(100)
			Italy	(101)
			Slovakia	(102)
			France	(102)
			Italy	(103)
			Japan	(98)
	Turtle dove	*Streptopelia turtur*	Bulgaria	(99)
	Wood-pigeon	*Columba palumbus*	Bulgaria	(99)
Corvidae	Crow	*Corvus spp*.	Bulgaria	(99)
			Japan	(98)
	Carrion crow	*Corvus corone*	Japan	(98)
	Hooded crow	*Corvus cornix*	Russia	(96)
	Magpie	*Pica spp*.	Bulgaria	(99)
	Raven	*Corvus corax*	Bulgaria	(99)
	Rook	*Corvus frugileus*	Russia	(104)
Hirundinidae	Barn swallow	*Hirundo rustica*	Czechoslovakia	(100)
Laridae	Black-headed gull	*Chroicocephalus ridibundus*	Russia	(105)
				(96)
	Common gull	*Larus canus*	Russia	(105)
	Common tern	*Sterna hirundo*	Russia	(105)
	White-winged tern	*Chlidonias leucopterus*	Russia	(105)
Motacillidae	Western yellow wagtail	*Motacilla flava*	Bulgaria	(96)
	White wagtail	*Motacilla alba*	Czechoslovakia	(100)
			Russia	(96)
Muscicapidae	Redstart	*Phoenicurus phoenicurus*	Czechoslovakia	(100)
	Thrush nightingale	*Luscinia luscinia*	Bulgaria	(96)
Passeridae	House sparrow	*Passer domesticus*	Russia	(96)
	Italian sparrow	*Passer italiae*	Italy	(103)
Pelecanidae	Great white pelican	*Pelecanus onocrotalus*	Bulgaria	(96)
Phasianidae	Common quail	*Coturnix coturnix*	Japan	(98)
			Russia	(96)
	Hens	*Gallus gallus domesticus*	Czechoslovakia	(100)
			Bulgaria	(99)
	Japanese quail	*Coturnix japonica*	Japan	(98)
	Pheasant	*Phasianus colchicus*	Bulgaria	(99)
	Turkey	*Meleagris spp*.	Czechoslovakia	(100)
Phylloscopidae	Willow warbler	*Phylloscopus trochilus*	Bulgaria	(96)
Psittaculidae	Eclectus parrot	*Eclectus roratus*	USA	(106)
Scolopacidae	Wood sandpiper	*Tringa graleola*	Bulgaria	(96)
Sturnidae	Common starling	*Sturnus vulgaris*	Russia	(96)
Turdidae	Common blackbird	*Turdus merula*	Russia	(96)
			Bulgaria	(96)
	Fieldfare	*Turdus pilaris*	Russia	(96)
***Coxiella burnetii infection in reptiles***				
Emydidae	Blanding's turtle	*Emydoidea blandingii*	USA	(107)
	Ornate box turtle	*Terrapene ornata*		
	Painted turtle	*Chrysemys picta*		
Geoemydidae	Roofed turtles	*Batagur and Pangshura* spp. *(formerly in genus Kachuga)*	India	(108)
Colubridae	Chinese ratsnake	*Ptyas korros*	India	(108)
	Grass snake	*Natrix natrix*		
Pythonidae	Indian python	*Python molurus*	India	(108)
***Coxiella burnetii infection in terrestrial mammals***				
Bovidae	Alpine chamois	*Rupicapra rubicapra*	France	(109)
	Alpine ibex	*Capra ibex*	Switzerland	(110)
	Bighorn sheep	*Ovis canadensis*	USA	(111)
	Cuvier's gazelle	*Gazella cuvieri*	Europe	(112)
	Dama gazelle	*Nanger dama*	UAE	(113)
			Europe	(112)
	Mouflon	*Ovis orientalis*	Spain	(114)
			Czech Republic	(115)
			Cyprus	(116)
				(117)
	Muskox	*Ovibos moschatus*	USA	(118)
	Sable antelope	*Hippotragus niger*	Portugal	(119)
	Saiga antelope	*Saiga tatarica*	Kazakhstan	(120)
	Spanish ibex	*Capra pyrenaica*	Spain	(121)
	Waterbuck	*Kobus ellipsiprymnus*	Portugal	(119)
	Yak	*Bos mutus*	China	(122)
Cervidae	Black-tailed deer	*Odocoileus hemionus colombianus*	USA	(123)
	California mule deer	*Odocoileus hemionus californicus*	USA	(123)
	Fallow deer	*Dama dama*	Czech Republic	(115)
			Italy	(124)
			Spain	(121)
			Czech Republic	(115)
			Slovakia	(125)
			Spain	(121)
	Red deer	*Cervus elaphus*	Spain	(94)
				(126)
				(114)
			France	(127)
			Italy	(128)
			Hungary	(129)
	Rocky mountain mule deer	*Odocoileus hemionus. hemionus*	USA	(123)
	Roe deer	*Capreolus capreolus*	France	(130)
				(131)
				(127)
				(132)
			Netherlands	(133)
			Czech Republic	(115)
			Slovakia	(95)
			Spain	(94)
	Sika deer	*Cervus nippon*	Japan	(134)
	White-tailed deer	*Odocoileus virginianus*	Canada	(135)
			USA	(136)
Suidae	Eurasian wild boar	*Sus scrofa*	Czech Republic	(115)
			Spain	(137)
				(95)
Leporidae	European hare	*Lepus europaeus*	Cyprus	(116)
			Spain	(95)
	European rabbit	*Oryctolagus cuniculus*	Spain	(95)
				(76)
				(70)
			Australia	(68)
	Japanese hare	*Lepus brachyurus*	Japan	(134)
Cricetidae	Bank vole	*Myodes glareolus*	UK	(88)
	Cursor Grass Mouse	*Akodon cursor*	Brazil	(138)
	Delta Pygmy Rice Rat	*Oligoryzomys nigripes*	Brazil	(138)
	Field vole	*Microtus agrestis*	UK	(88)
	Red vole	*Myodes rutilus*	Russia/China	(139)
	Reed vole	*Microtus fortis*	Russia/China	(139)
	The Atlantic hucicudo	*Oxymycterus dasytrichus*	Brazil	(138)
Muridae	Black rat	*Rattus rattus*	Netherlands	(140)
	Brown rat	*Rattus norvegicus*	Russia/China	(139)
			Germany	(141)
			Netherl.	(140)
	House mouse	*Mus musculus*	Spain	(42)
			Brazil	(138)
	Large Japanese Field Mouse	*Apodemus speciosus*	Russia/China	(139)
	Long-tailed field mouse	*Apodemus sylvaticus*	Italy	(142)
			Spain	(42)
			UK	(88)
	Striped Field Mouse	*Apodemus agrarius*	Russia/China	(139)
Sciuridae	American red squirrel	*Tamiasciurus hudsonicus*	Canada	(143)
	Carolina flying squirrel	*Glaucomys sabrinus*	Canada	(143)
	Siberian Chipmunk	*Tamias sibiricus*	Russia/China	(139)
Canidae	Coyote	*Canis latrans*	USA	(144)
	Red fox	*Vulpes vulpes*	Cyprus	(116)
			Spain	(145)
			UK	(88)
Erinaceidae	Amur hedgehog	*Erinaceus amurensis*	China	(146)
	North African Hedgehog	*Atelerix algirus*	Tunisia	(147)
Felidae	Jaguar	*Panthera onca*	Brazil	(148)
	Wild cat	*Felis silvestris*	Spain	(121)
Macropodidae	Western grey kangaroo	*Macropus fuliginosus*	Australia	(149)
Viverridae	Common genet	*Genetta genetta*	Spain	(145)
***Coxiella burnetii infection in marine mammals***				
Mustelidae	Sea otter	*Enhydra lutris*	USA	(150)
Otariidae	Northern fur seal	*Callorhinus ursinus*	USA	(151)
				(152)
	Steller sea lion	*Eumetopias jubatus*	USA	(151)
				(153)
Phocidae	Harbor seal	*Phoca vitulina richardsi*	USA	(153)

The pathogenesis of *C. burnetii* infection in birds is not well-defined. Previous studies observed *Coxiella* persistence in birds with (polyorganous lesions resulting in mortality) and without clinical signs (101, 106, 154).

In reptiles, two earlier studies have identified *C. burnetii* in India, where two tortoises, snakes and skinks were seropositive for *C. burnetii* (108, 155). A recent study examined turtles in Illinois and Wisconsin, USA, for *C. burnetii* using qPCR, and 5 out of 605 turtles yielded positive results for *C. burnetii* (107). Furthermore, *C. burnetii* has been detected in various reptilian ticks, including tortoise tick *H. aegyptium* from Romania (156), *Amblyomma exornatum* from Guinea Bissau, *A. nuttalli* from Ghana, and *A. variegatum* in Africa (157).

In mammals, *C. burnetii* infection has been detected in a broad range of species. In cervids, *C. burnetii* has been reported in black-tailed deer (*Odocoileus hemionus colombianus*), California mule deer *(O. h. californicus)*, Rocky Mountain mule deer (*O. h. hemionus*), and white-tailed deer *(Odocoileus virginianus)* in Canada and US (123, 135, 136). For European cervids, the infection has been documented in fallow deer (*Dama dama*), red deer (*Cervus elaphus*), and roe deer (*Capreolus capreolus*) (94, 95, 114, 115, 121, 124–133). The serological evidence of the infection was also reported in Sika deer (*Cervus arbor*) in Japan (134). Furthermore, the presence of *C. burnetii* has been detected in wild boars, hares, and many rodent species; including Eurasian wild boars (*Sus scrofa*) (95, 115, 137), European hares (*Lepus europaeus*) (95, 116), European rabbit (*Oryctolagus cuniculus*) (68, 70, 76), Japanese hare (*Lepus brachyurus*) (134), cursor grass mouse (*Akodon cursor*) (138, 158), bank vole (*Myodes glareolus*) (88), delta pygmy rice rat (*Oligoryzomys nigripes*) (138), the Atlantic forest hocicudo (*Oxymycterus dasytrichus*) (138), field vole (*Microtus agrestis*) (88), reed vole (*Microtus fortis*) (139), red vole (*Myodes rutilus*) (139), black rat (*Rattus rattus*) (140), brown rat (*Rattus norvegicus*) (139–141), house mouse (*Mus musculus*) (42, 138), long-tailed field mouse (*Apodemus sylvaticus*) (42, 88, 142), large Japanese field mouse (*Apodemus speciosus*) (139), striped field mouse (*Apodemus agrarius*) (139), American red squirrel (*Tamiasciurus hudsonicus*) (143), Carolina flying squirrel (*Glaucomys sabrinus*) (143), and Siberian chipmunk (*Tamias sibiricus*) (139). Rodents are considered to be significant reservoirs of infection in the domestic cycle of *C. burnetii* (159), and several rodent species have been found to be a source of livestock coxiellosis (140, 160).

Additionally, *C. burnetii* has been implicated in reproductive losses in captive exotic ungulates, including waterbuck (*Kobus ellipsiprymnus*) (119), sable antelope (*Hippotragus nige*r) (119), and many gazelles, such as arbor gazelle (*Gazella arbor neglecta*), dama gazelle (*Nanger dama mhorr*) and Cuvier's gazelle (*Gazella cuvieri*) (112, 113). Reproductive disorders caused by *C. burnetii* in endangered species such as exotic ungulates that are bred in captivity for conservation programs may be of critical threat and pose a risk for the programs (113). Other bovids reported to be exposed to/infected by *C. burnetii* are Alpine ibex (*Capra ibex*) (110), Bighorn sheep (*Ovis arborsis*) (111), Alpine chamois (*Rupicapra rupicapra*) (109), Spanish ibex (*Capra pyrenaica*) (121), mouflon (*Ovis orientalis*) (114–117), muskox (*Ovibos moschatus*) (118), saiga antelope (*Saiga tatarica*) (120), and yak (*Bos mutus*) (122).

Apart from terrestrial mammals, *C. burnetii* has also been detected in marine wildlife—e.g., sea otter (*Enhydra lutris*) (150), northern fur seal (*Callorhinus ursinus*) (151, 152), steller sea lion (*Eumetopias jubatus*) (151, 153, 161), and harbor seal (*Phoca vitulina richardsi*) (153).

Other mammals that have been shown to harbor *C. burnetii* are coyote (*Canis latrans*) (144), red fox (*Vulpes vulpes*) (88, 116, 145), jaguar (*Panthera onca*) (148), wild cat (*Felis silvestris*) (121), common genet (*Genetta genetta*) (145), western grey kangaroo (*Macropus fuliginosus*) (149), North African hedgehog (*Atelerix algrus*) (147), and Amur hedgehog (*Erinaceus amurensis*) (146).

Coxiellosis causes similar clinical outcomes and pathologies in wild animals as it does in domestic animals (12). Placentitis is one of the most common lesions identified in wild animals with coxiellosis which have been observed in dama gazelle (113), Steller sea lion (161), and Pacific harbor seal (162).

## Disease control

Preventive veterinary practices are critical in the control of coxiellosis. Two methods are available to control coxiellosis in animals: vaccination and antibiotic treatment.

Vaccination is one of the most effective management strategies to reduce abortion rates and spread of the bacterium. WOHA recommends only the administration of vaccines containing or prepared from phase I *C. burnetii* as it has been scientifically showed that the full-length phase I lipopolysaccharide is the protective antigen of *C. burnetii* (163). Vaccines prevent successive transmission to healthy individuals and humans, and reduce but do not eliminate shedding of the bacterium (164–166). Two vaccines against *C. burnetii* are currently commercially available for veterinary use in many regions of the world. The first one is an inactivated bivalent vaccine developed from *Chlamydia abortus* and phase II *C. burnetii* (Chlamyvax^®^, Mérial, Lyon, France), indicated for use in sheep and goats. The second one is an inactivated non-adjuvanted phase I *C. burnetii* antigen Nine Mile strain vaccine (Coxevac^®^, CEVA Santé Animale, Libourne, France) recommended for use in goats and cattle (167).

Antibiotic treatment is another available option to control coxiellosis in animals. Pregnant animals might have a decrease in abortion rates and *C. burnetii* shedding by receiving antibiotic treatment with oxytetracycline (20 mg/kg) during the last trimester of pregnancy (168). However, antibiotic treatment of animals is not recommended since the effect of the treatment is not sufficiently demonstrated and proportionate use of antibiotics is required to avoid microbial resistance (8).

## Conclusion

Q fever is a significant zoonotic disease worldwide that affects both public and veterinary health, as well as has a detrimental socioeconomic impact on livestock industry. In view of the threats related to this disease, a thorough understanding of transmission routes and potential sources of infection is crucial. *Coxiella burnetii* has been detected in various hosts, including humans, domestic and wild animals, pets, birds, and arthropods. Ticks are considered as vectors of *C. burnetii* and may pose a risk for infection of animals and humans. However, further field studies should be implemented to assess the role of the ticks as vectors for *C. burnetii* under natural conditions. Understanding their role can help us to develop and/or improve vector control strategies that would lead to decrease of *C. burnetii* risk. It is also noteworthy that our understanding about *C. burnetii* infection patterns in ticks and role of ticks as possible *C. burnetii* vectors are limited since CLEs are likely to have been mistakenly identified as *C. burnetii* in many studies. The development of the diagnostic tests to distinguish between *C. burnetii* and CLEs to improve our understanding of Q fever epidemiology should also be a key area of future research.

Another point of contention is the role of wildlife—livestock—human interactions which should be further investigated. It is important to develop effective preventive and control strategies using on evidence-based “One Health” approach. In the context of inadequate biosafety controls implemented in the wildlife—livestock—human interface, the possibility of a high rate of transmission of the zoonotic pathogens, including *C. burnetii*, at these interfaces cannot be precluded.

Further studies are also required to better understand the pathogenicity of *C. burnetii* for its arthropod and wild hosts. Research about possible routes of transmission of *C. burnetii* between different host should not be omitted as well. Finally, we should focus also on research evaluating the pathogenicity of CLEs for humans and other mammals or at least their ability to infect them.

## Author contributions

SSC and JČ: conceptualization and writing—review and editing. SSC: writing—original draft preparation and visualization. JČ: supervision. All authors contributed to the article and approved the submitted version.

## Conflict of interest

The authors declare that the research was conducted in the absence of any commercial or financial relationships that could be construed as a potential conflict of interest.

## Publisher's note

All claims expressed in this article are solely those of the authors and do not necessarily represent those of their affiliated organizations, or those of the publisher, the editors and the reviewers. Any product that may be evaluated in this article, or claim that may be made by its manufacturer, is not guaranteed or endorsed by the publisher.

## References

[1] Babudieri B. Q fever: a zoonosis. Adv Vet Sci. (1959) 5:81–154.

[2] Rathish B Pillay R Wilson A Pillay VV. Comprehensive review of bioterrorism. In: StatPearls. Treasure Island, FL: StatPearls Publishing (2022). 34033376

[3] Pouquet M Bareille N Guatteo R Moret L Beaudeau F. *Coxiella burnetii* infection in humans: to what extent do cattle in infected areas free from small ruminants play a role? Epidemiol Infect. (2020) 148:e232. 10.1017/S0950268820001880 32843112 PMC7582459

[4] Pexara A Solomakos N Govaris A. Q fever and seroprevalence of *Coxiella burnetii* in domestic ruminants. Vet Ital. (2018) 54:265–79. 10.12834/VetIt.1113.6046.3 30681125

[5] Devaux CA Osman IO Million M Raoult D. *Coxiella burnetii* in dromedary camels (*Camelus dromedarius*): a possible threat for humans and livestock in North Africa and the near and middle east? Front Vet Sci. (2020) 7:558481. 10.3389/fvets.2020.558481 33251255 PMC7674558

[6] Hilbink F Penrose M Kovacova E Kazar J. Q. fever is absent from New Zealand. Int J Epidemiol. (1993) 22:945–9. 10.1093/ije/22.5.945 8282477

[7] Angelakis E Raoult D. Q. fever. Vet Microbiol. (2010) 140:297–309. 10.1016/j.vetmic.2009.07.016 19875249

[8] EFSA. Scientific opinion on Q fever. EFSA J. (2010) 8:1595. 10.2903/j.efsa.2010.1595

[9] Vanderburg S Rubach MP Halliday JEB Cleaveland S Reddy EA Crump JA. Epidemiology of *Coxiella burnetii* infection in Africa: a onehealth systematic review. PLoS Negl Trop Dis. (2014) 8:e2787. 10.1371/journal.pntd.0002787 24722554 PMC3983093

[10] Raoult D Marrie T Mege J. Natural history and pathophysiology of Q fever. Lancet Infect Dis. (2005) 5:219–26. 10.1016/S1473-3099(05)70052-9 15792739

[11] Sánchez J Souriau A Buendía AJ Arricau-Bouvery N Martínez CM Salinas J. Experimental *Coxiella burnetii* infection in pregnant goats: a histopathological and immunohistochemical study. J Comp Pathol. (2006) 135:108–15. 10.1016/j.jcpa.2006.06.003 16997003

[12] González-Barrio D Ruiz-Fons F. *Coxiella burnetii* in wild mammals: a systematic review. Transbound Emerg Dis. (2019) 66:662–71. 10.1111/tbed.13085 30506629

[13] Duron O Sidi Boumedine K Rousset E Moutailler S Jourdain E. The importance of ticks in Q fever transmission: what has (and has not) been demonstrated? Trends Parasitol. (2015) 31:536–52. 10.1016/j.pt.2015.06.014 26458781

[14] Ebani VV Mancianti F. Potential role of birds in the epidemiology of *Coxiella burnetii, Coxiella*-like agents and *Hepatozoon spp*. *Pathogens*. (2022) 11:298. 10.3390/pathogens11030298 35335622 PMC8954922

[15] Abdel-Moein KA Zaher HM. Parturient cat as a potential reservoir for *Coxiella burnetii*: a hidden threat to pet owners. Vector Borne Zoonotic Dis. (2021) 21:264–8. 10.1089/vbz.2020.2714 33434106

[16] Shapiro A Bosward K Mathews K Vincent G Stenos J Tadepalli M Norris J. Molecular detection of *Coxiella burnetii* in raw meat intended for pet consumption. Zoonoses Public Health. (2020) 67:443– 452. 10.1111/zph.12707 32347659

[17] Maurin M Raoult D. Q. fever. Clin Microbiol Rev. (1999) 12:518–53. 10.1128/CMR.12.4.518 10515901 PMC88923

[18] Derrick EH. “Q” fever, a new fever entity: clinical features, diagnosis and laboratory investigation. Med J Aust. (1937) 2:281–99. 10.5694/j.1326-5377.1937.tb43743.x 6622891

[19] Davis GE Cox HR Parker RR Dyer RE. A filter-passing infectious agent isolated from ticks. Public Health Rep. (1938) 53:2259. 10.2307/458274619315693

[20] Smith D Derrick E. Studies in the epidemiology of Q fever. Aust J Exp Biol Med Sci. (1940) 18:1–8. 10.1038/icb.1940.1

[21] Shipman M Lubick K Fouchard D Gurram R Grieco P Jutila M. Proteomic and systems biology analysis of the monocyte response to *Coxiella burnetii* infection. PLoS ONE. (2013) 8:e69558. 10.1371/journal.pone.0069558 23990884 PMC3749201

[22] Hussain S Perveen N Hussain A Song B Aziz MU Zeb J. The symbiotic continuum within ticks: opportunities for disease control. Front Microbiol. (2022) 13:854803. 10.3389/fmicb.2022.854803 35369485 PMC8969565

[23] Jourdain E Duron O Barry S González-Acuña D Sidi-Boumedine K. Molecular methods routinely used to detect *Coxiella burnetii* in ticks cross-react with *Coxiella*-like bacteria. Infect Ecol Epidemiol. (2015) 5:29230. 10.3402/iee.v5.29230 26609691 PMC4660934

[24] Duron O Noël V McCoy KD Bonazzi M Sidi-Boumedine K Morel O. The recent evolution of a maternally-inherited endosymbiont of ticks led to the emergence of the Q fever pathogen, *Coxiella burnetii*. *PLoS Pathog*. (2015) 11:e1004892. 10.1371/journal.ppat.1004892 25978383 PMC4433120

[25] Eldin C Mélenotte C Mediannikov O Ghigo E Million M Edouard S. From Q fever to *Coxiella burnetii* infection: a paradigm change. Clin Microbiol Rev. (2017) 30:115–90. 10.1128/CMR.00045-16 27856520 PMC5217791

[26] van Engelen E Schotten N Schimmer B Hautvast JLA van Schaik G van Duijnhoven Y. Prevalence and risk factors for *Coxiella burnetii* (Q fever) in dutch dairy cattle herds based on bulk tank milk testing. Prev Vet Med. (2014) 117:103–9. 10.1016/j.prevetmed.2014.08.016 25239684

[27] Cantas H Muwonge A Sareyyupoglu B Yardimci H Skjerve E. Q fever abortions in ruminants and associated on-farm risk factors in northern Cyprus. BMC Vet Res. (2011) 7:13. 10.1186/1746-6148-7-13 21414196 PMC3070639

[28] Asadi J Khalili M Kafi M Ansari-Lari M Hosseini SM. Risk factors of Q fever in sheep and goat flocks with history of abortion. Comp Clin Path. (2014) 23:625–30. 10.1007/s00580-012-1661-9

[29] Benaissa MH Ansel S Mohamed-Cherif A Benfodil K Khelef D Youngs CR. Seroprevalence and risk factors for *Coxiella burnetii*, the causative agent of Q fever in the dromedary camel (*Camelus dromedarius*) population in Algeria. Onderstepoort J Vet Res. (2017) 84:e1–7. 10.4102/ojvr.v84i1.1461 28893076 PMC6238797

[30] DráŽovská M Prokeš M Vojtek B MojŽišová J Ondrejková A Korytár L. First serological record of *Coxiella burnetii* infection in the equine population of Slovakia. Biologia. (2022) 77:1645–9. 10.1007/s11756-021-00898-4

[31] Kazar J. *Coxiella burnetii* infection. Ann N Y Acad Sci. (2005) 1063:105–14. 10.1196/annals.1355.018 16481501

[32] Koka H Sang R Kutima HL Musila L. *Coxiella burnetii* detected in tick samples from pastoral communities in Kenya. Biomed Res Int. (2018) 2018:8158102. 10.1155/2018/8158102 30105251 PMC6076967

[33] Lang GH. Q fever: an emerging public health concern in Canada. Can J Vet Res. (1989) 53:1–6. 2644004 PMC1255503

[34] Philip CB. Observations on experimental Q fever. J Parasitol. (1948) 34:457–64. 10.2307/3273312 18099928

[35] Abdullah H Hussein HA El-Razik KA Barakat AM Soliman YA. Q fever: a neglected disease of camels in Giza and Cairo Provinces, Egypt. Vet World. (2019) 12:1945–50. 10.14202/vetworld.2019.1945-1950 32095045 PMC6989333

[36] Körner S Makert GR Ulbert S Pfeffer M Mertens-Scholz K. The prevalence of *Coxiella burnetii* in hard ticks in Europe and their role in Q fever transmission revisited-a systematic review. Front Vet Sci. (2021) 8:655715. 10.3389/fvets.2021.655715 33981744 PMC8109271

[37] Sprong H Tijsse-Klasen E Langelaar M de Bruin A Fonville M Gassner F. Prevalence of *Coxiella burnetii* in ticks after a large outbreak of Q fever. Zoonoses Public Health. (2012) 59:69–75. 10.1111/j.1863-2378.2011.01421.x 21824373

[38] Wallménius K Pettersson JH-O Jaenson TGT Nilsson K. Prevalence of *Rickettsia spp, Anaplasma phagocytophilum*, and *Coxiella burnetii* in adult Ixodes ricinus ticks from 29 study areas in central and southern Sweden. Ticks Tick Borne Dis. (2012) 3:100–6. 10.1016/j.ttbdis.2011.11.003 22487426

[39] Reháček J Úrvölgyi J Kocianová E Sekeyová Z Vavrekova M Kováčová E. Extensive examination of different tick species for infestation with *Coxiella burnetii* in Slovakia. Eur J Epidemiol. (1991) 7:299–303. 10.1007/BF00145682 1884784

[40] Špitalská E Kocianová E. Detection of *Coxiella burnetii* in ticks collected in Slovakia and Hungary. Eur J Epidemiol. (2002) 18:263–6. 10.1023/A:1023330222657 12800953

[41] Hildebrandt A Straube E Neubauer H Schmoock G. *Coxiella burnetii* and coinfections in *Ixodes ricinus* ticks in central Germany. Vector Borne Zoonotic Dis. (2011) 11:1205–7. 10.1089/vbz.2010.0180 21142964

[42] Barandika JF Hurtado A García-Sanmartín J Juste RA Anda P García-Pérez AL. Prevalence of tick- borne zoonotic bacteria in questing adult ticks from Northern Spain. Vector Borne Zoonotic Dis. (2008) 8:829–36. 10.1089/vbz.2008.0023 18759563

[43] Reháček J Kaaserer B Úrvölgyi J Lukáčová M Kováčová E Kocianová E. Isolation of *Coxiella burnetii* and of an unknown rickettsial organism from *Ixodes ricinus* ticks collected in Austria. Eur J Epidemiol. (1994) 10:719–23. 10.1007/BF01719288 7545588

[44] Rodolakis A. Q Fever in dairy animals. Ann N Y Acad Sci. (2009) 1166:90–3. 10.1111/j.1749-6632.2009.04511.x 19538267

[45] Rodolakis A Berri M Héchard C Caudron C Souriau A Bodier CC. Comparison of *Coxiella burnetii* shedding in milk of dairy bovine, caprine, and ovine herds. J Dairy Sci. (2007) 90:5352–60. 10.3168/jds.2006-815 18024725

[46] Plummer PJ McClure JT Menzies P Morley PS van den Brom R van Metre DC. Management of *Coxiella burnetii* infection in livestock populations and the associated zoonotic risk: a consensus statement. J Vet Intern Med. (2018) 32:1481–94. 10.1111/jvim.15229 30084178 PMC6189356

[47] Szymańska-Czerwińska M Jodełko A Zareba-Marchewka K Niemczuk K. Shedding and genetic diversity of *Coxiella burnetii* in polish dairy cattle. PLoS ONE. (2019) 14:e0210244. 10.1371/journal.pone.0210244 30629637 PMC6328121

[48] Brom RV den Engelen E van Roest HIJ Hoek W van der Vellema P. *Coxiella burnetii* infections in sheep or goats: an opinionated review. Vet Microbiol. (2015) 181:119–29. 10.1016/j.vetmic.2015.07.011 26315774

[49] Tilburg JJHC Roest HJIJ Nabuurs-Franssen MH Horrevorts AM Klaassen CHW. Genotyping reveals the presence of a predominant genotype of *Coxiella burnetii* in consumer milk products. J Clin Microbiol. (2012) 50:2156–8. 10.1128/JCM.06831-11 22495560 PMC3372143

[50] Archer BN Hallahan C Stanley P Seward K Lesjak M Hope K. Atypical outbreak of Q fever affecting low-risk residents of a remote rural town in New South Wales. Commun Dis Intell Q Rep. (2017) 41:E125–33. 28899307 10.33321/cdi.2017.41.17

[51] Buhariwalla F Cann B Marrie TJ. A dog-related outbreak of Q fever. Clin Infect Dis. (1996) 23:753–5. 10.1093/clinids/23.4.753 8909839

[52] Pinsky RL Fishbein DB Greene CR Gensheimer KF. An outbreak of cat-associated Q fever in the United States. J Infect Dis. (1991) 164:202–4. 10.1093/infdis/164.1.202 2056206

[53] Hornok S Dénes B Meli ML Tánczos B Fekete L Gyuranecz M. Non-pet dogs as sentinels and potential synanthropic reservoirs of tick-borne and zoonotic bacteria. Vet Microbiol. (2013) 167:700–3. 10.1016/j.vetmic.2013.08.011 24021884

[54] Laughlin T Waag D Williams J Marrie T. Q fever: from deer to dog to man. Lancet. (1991) 337:676–7. 10.1016/0140-6736(91)92494-M 1672016

[55] Marrie TJ Langille D Papukna V Yates L. Truckin' pneumonia—an outbreak of Q fever in a truck repair plant probably due to aerosols from clothing contaminated by contact with newborn kittens. Epidemiol Infect. (1989) 102:119–27. 10.1017/S0950268800029757 2917613 PMC2249319

[56] Marrie TJ Durant H Williams JC Mintz E Waag DM. Exposure to parturient cats: a risk factor for acquisition of Q fever in maritime Canada. J Infect Dis. (1988) 158:101–8. 10.1093/infdis/158.1.101 3392409

[57] Norris JM Bosward KL Heller J. Q fever: pets, vets and validating tests. Microbiol Aust. (2013) 34:186. 10.1071/MA13064

[58] Browne AS Fèvre EM Kinnaird M Muloi DM Wang CA Larsen PS. Serosurvey of *Coxiella burnetii* (Q fever) in dromedary camels (*Camelus dromedarius*) in Laikipia County, Kenya. Zoonoses Public Health. (2017) 64:543–9. 10.1111/zph.12337 28176495 PMC5655913

[59] Seo M-G Ouh I-O Lee S-H Kwak D. Detection and genotyping of *Coxiella burnetii* in pigs, South Korea, 2014–2015. Emerg Infect Dis. (2016) 22:2192–5. 10.3201/eid2212.161236 27869590 PMC5189167

[60] Klemmer J Njeru J Emam A El-Sayed A Moawad AA Henning K. Q fever in Egypt: epidemiological survey of *Coxiella burnetii* specific antibodies in cattle, buffaloes, sheep, goats and camels. PLoS ONE. (2018) 13:e0192188. 10.1371/journal.pone.0192188 29466380 PMC5821454

[61] Hussein MF Alshaikh MA Al-Jumaah RS GarelNabi A Al-Khalifa I Mohammed OB. The Arabian camel (*Camelus dromedariu*s) as a major reservoir of Q fever in Saudi Arabia. Comp Clin Path. (2015) 24:887–92. 10.1007/s00580-014-2002-y

[62] Bellabidi M Benaissa MH Bissati-Bouafia S Harrat Z Brahmi K Kernif T. *Coxiella burnetii* in camels (*Camelus dromedarius*) from Algeria: Seroprevalence, molecular characterization, and ticks (Acari: Ixodidae) vectors. Acta Trop. (2020) 206:105443. 10.1016/j.actatropica.2020.105443 32173315

[63] Fu M He P OuYang X Yu Y Wen B Zhou D. Novel genotypes of *Coxiella burnetii* circulating in rats in Yunnan Province, China. BMC Vet Res. (2022) 18:204. 10.1186/s12917-022-03310-8 35624449 PMC9137106

[64] Webster JP Lloyd G Macdonald DW Q. fever (*Coxiella burnetii*) reservoir in wild brown rat (*Rattus norvegicus*) populations in the UK. Parasitology. (1995) 110:31–5. 10.1017/S0031182000081014 7845709

[65] Keshavamurthy R Singh BB Kalambhe DG Aulakh RS Dhand NK. Prevalence of *Coxiella burnetii* in cattle and buffalo populations in Punjab, India. Prev Vet Med. (2019) 166:16–20. 10.1016/j.prevetmed.2019.03.003 30935501

[66] Khademi P Ownagh A Mardani K Khalili M. Prevalence of *Coxiella burnetii* in milk collected from buffalo (water buffalo) and cattle dairy farms in Northwest of Iran. Comp Immunol Microbiol Infect Dis. (2019) 67:101368. 10.1016/j.cimid.2019.101368 31627037

[67] Kidsin K Panjai D Boonmar S. The first report of seroprevalence of Q fever in water buffaloes (*Bubalus bubalis*) in Phatthalung, Thailand. Vet World. (2021) 14:2574–8. 10.14202/vetworld.2021.2574-2578 34840480 PMC8613777

[68] Caraguel C Bassett S González-Barrio D Elsworth P Chaber A-L. Comparison of three serological tests for the detection of *Coxiella burnetii* specific antibodies in European wild rabbits. BMC Vet Res. (2020) 16:315. 10.1186/s12917-020-02526-w 32859195 PMC7456029

[69] Bolaños-Rivero M Carranza-Rodríguez C Rodríguez NF Gutiérrez C Pérez-Arellano J-L. Detection of *Coxiella burnetii* DNA in peridomestic and wild animals and ticks in an endemic region (Canary Islands, Spain). Vector Borne Zoonotic Dis. (2017) 17:630–4. 10.1089/vbz.2017.2120 28759337

[70] Sánchez M Valcárcel F González J González MG Martín-Hernández R Tercero JM. Seasonality of *Coxiella burnetii* among wild rabbits (*Oryctolagus cuniculus*) and the *Hyalomma lusitanicum* (Acari: Ixodidae) in a meso-mediterranean ecosystem. Pathogens. (2021) 11:36. 10.3390/pathogens11010036 35055984 PMC8781871

[71] Leon A Richard E Fortier C Laugier C Fortier G Pronost S. Molecular detection of *Coxiella burnetii* and *Neospora caninum* in equine aborted foetuses and neonates. Prev Vet Med. (2012) 104:179–83. 10.1016/j.prevetmed.2011.11.001 22130310

[72] Akter R Legione A Sansom FM El-Hage CM Hartley CA Gilkerson JR. Detection of *Coxiella burnetii* and equine herpesvirus 1, but not *Leptospira* spp. or *Toxoplasma gondii*, in cases of equine abortion in Australia - a 25 year retrospective study. PLoS ONE. (2020) 15:e0233100. 10.1371/journal.pone.0233100 32453753 PMC7250447

[73] Lubova VA Leonova GN Shutikova AL Bondarenko EI. Indication Q fever pathogen in the south of Far east. Klin Lab Diagn. (2020) 65:724–8. 10.18821/0869-2084-2020-65-11-724-728 33301664

[74] Desjardins I Joulié A Pradier S Lecollinet S Beck C Vial L. Seroprevalence of horses to *Coxiella burnetii* in an Q fever endemic area. Vet Microbiol. (2018) 215:49–56. 10.1016/j.vetmic.2017.11.012 29426406

[75] Li J Li Y Moumouni PFA Lee S-H Galon EM Tumwebaze MA. First description of *Coxiella burnetii* and *Rickettsia sp*p infection and molecular detection of piroplasma co-infecting horses in Xinjiang Uygur Autonomous Region, China. Parasitol Int. (2020) 76:102028. 10.1016/j.parint.2019.102028 31759172

[76] González-Barrio D Maio E Vieira-Pinto M Ruiz-Fons F. European rabbits as reservoir for *Coxiella burnetii*. *Emerg Infect Dis*. (2015) 21:1055–8. 10.3201/eid2106.141537 25988670 PMC4451900

[77] Marmion BP Stoker MGP. Epidemiology of Q fever in great britain. BMJ. (1958) 2:809–16. 10.1136/bmj.2.5100.809 13572912 PMC2026473

[78] Gossner C Danielson N Gervelmeyer A Berthe F Faye B Kaasik Aaslav K. Human–dromedary camel interactions and the risk of acquiring zoonotic middle east respiratory syndrome coronavirus infection. Zoonoses Public Health. (2016) 63:1–9. 10.1111/zph.12171 25545147 PMC7165574

[79] Zhu S Zimmerman D Deem SL. A review of zoonotic pathogens of dromedary camels. Ecohealth. (2019) 16:356–77. 10.1007/s10393-019-01413-7 31140075 PMC7087575

[80] Selmi R Mamlouk A ben Yahia H Abdelaali H ben Said M Sellami K. *Coxiella burnetii* in Tunisian dromedary camels (*Camelus dromedarius*): Seroprevalence, associated risk factors and seasonal dynamics. Acta Trop. (2018) 188:234–9. 10.1016/j.actatropica.2018.09.008 30219555

[81] Agerholm JS Jensen TK Agger JF Engelsma MY Roest HIJ. Presence of *Coxiella burnetii* DNA in inflamed bovine cardiac valves. BMC Vet Res. (2017) 13:69. 10.1186/s12917-017-0988-5 28274243 PMC5343293

[82] de Biase D Costagliola A del Piero F di Palo R Coronati D Galiero G. *Coxiella burnetii* in infertile dairy cattle with chronic endometritis. Vet Pathol. (2018) 55:539–42. 10.1177/0300985818760376 29566608

[83] Russell-Lodrigue KE Zhang GQ McMurray DN Samuel JE. Clinical and pathologic changes in a guinea pig aerosol challenge model of acute Q fever. Infect Immun. (2006) 74:6085–91. 10.1128/IAI.00763-06 17057087 PMC1695512

[84] Russell-Lodrigue KE Andoh M Poels MWJ Shive HR Weeks BR Zhang GQ. *Coxiella burnetii* isolates cause genogroup-specific virulence in mouse and guinea pig models of acute Q fever. Infect Immun. (2009) 77:5640–50. 10.1128/IAI.00851-09 19786560 PMC2786457

[85] Ma GC Norris JM Mathews KO Chandra S Šlapeta J Bosward KL. New insights on the epidemiology of *Coxiella burnetii* in pet dogs and cats from New South Wales, Australia. Acta Trop. (2020) 205:105416. 10.1016/j.actatropica.2020.105416 32105667

[86] López-Olvera JR Vidal D Vicente J Pérez M Luján L Gortázar C. Serological survey of selected infectious diseases in mouflon (*Ovis aries musimon*) from south-central Spain. Eur J Wildl Res. (2009) 55:75–9. 10.1007/s10344-008-0215-6

[87] Kazimírová M Hamšíková Z Špitalská E Minichová L Mahríková L Caban R. Diverse tick-borne microorganisms identified in free-living ungulates in Slovakia. Parasit Vectors. (2018) 11:495. 10.1186/s13071-018-3068-1 30176908 PMC6122462

[88] Meredith AL Cleaveland SC Denwood MJ Brown JK Shaw DJ. *Coxiella burnetii* (Q-Fever) seroprevalence in prey and predators in the united kingdom: evaluation of infection in wild rodents, foxes and domestic cats using a modified ELISA. Transbound Emerg Dis. (2015) 62:639–49. 10.1111/tbed.12211 24479951

[89] González J González MG Valcárcel F Sánchez M Martín-Hernández R Tercero JM. Prevalence of *Coxiella burnetii* (Legionellales: Coxiellaceae) infection among wildlife species and the tick *Hyalomma lusitanicum* (Acari: Ixodidae) in a meso-mediterranean ecosystem. J Med Entomol. (2019) 57:551–6. 10.1093/jme/tjz169 31589748

[90] Bielawska-Drózd A Cieslik P Zakowska D Głowacka P Wlizło-Skowronek B Zieba P. Detection of *Coxiella burnetii* and *Francisella tularensis* in tissues of wild-living animals and in ticks of North-west Poland. Pol J Microbiol. (2018) 67:529–34. 10.21307/pjm-2018-059 30550240 PMC7256700

[91] Davoust B Marié J-L Pommier de Santi V Berenger J-M Edouard S Raoult D. Three-toed sloth as putative reservoir of *Coxiella burnetii*, Cayenne, French Guiana. Emerg Infect Dis. (2014) 20:1760–1. 10.3201/eid2010.140694 25271976 PMC4193280

[92] Stevenson S Gowardman J Tozer S Woods M. Life-threatening Q fever infection following exposure to kangaroos and wallabies. BMJ Case Rep. (2015) 2015:bcr2015210808. 10.1136/bcr-2015-210808 26385915 PMC4577632

[93] Arricau-Bouvery N Rodolakis A. Is Q fever an emerging or re-emerging zoonosis? Vet Res. (2005) 36:327–49. 10.1051/vetres:2005010 15845229

[94] Ruiz-Fons F Rodríguez Ó Torina A Naranjo V Gortázar C de la Fuente J. Prevalence of *Coxiella burnetti* infection in wild and farmed ungulates. Vet Microbiol. (2008) 126:282–6. 10.1016/j.vetmic.2007.06.020 17669603

[95] Astobiza I Barral M Ruiz-Fons F Barandika JF Gerrikagoitia X Hurtado A. Molecular investigation of the occurrence of *Coxiella burnetii* in wildlife and ticks in an endemic area. Vet Microbiol. (2011) 147:190–4. 10.1016/j.vetmic.2010.05.046 20580169

[96] Tokarevich NK Panferova YuA Freylikhman OA Blinova OV Medvedev SG Mironov SV. *Coxiella burnetii* in ticks and wild birds. Ticks Tick Borne Dis. (2019) 10:377–85. 10.1016/j.ttbdis.2018.11.020 30509727

[97] Ebani VV Nardoni S Giani M Rocchigiani G Archin T Altomonte I. Molecular survey on the occurrence of avian haemosporidia, *Coxiella burnetii* and *Francisella tularensis* in waterfowl from central Italy. Int J Parasitol Parasites Wildl. (2019) 10:87–92. 10.1016/j.ijppaw.2019.07.008 31384551 PMC6664032

[98] To H Sakai R Shirota K Kano C Abe S Sugimoto T. Coxiellosis in domestic and wild birds from Japan. J Wildl Dis. (1998) 34:310–6. 10.7589/0090-3558-34.2.310 9577778

[99] Martinov S. Contemporary state of the problem Q fever in Bulgaria. Biotechnol Biotechnol Equip. (2007) 21:353–61. 10.1080/13102818.2007.10817473

[100] Raska K Syucek L. Q fever in domestic and wild birds. Bull World Health Organ. (1956) 15:329–37. 13383368 PMC2538165

[101] Ebani VV Bertelloni F Mani P. Molecular survey on zoonotic tick-borne bacteria and chlamydiae in feral pigeons (*Columba livia domestica*). Asian Pac J Trop Med. (2016) 9:324–7. 10.1016/j.apjtm.2016.03.005 27086148

[102] Stein A Raoult D. Pigeon pneumonia in provence: a bird-borne Q fever outbreak. Clin Infect Dis. (1999) 29:617–20. 10.1086/598643 10530457

[103] Babudieri B Moscovici C. Experimental and natural infection of birds by *Coxiella burnetii*. *Nature*. (1952) 169:195–6. 10.1038/169195a0 14910721

[104] Basova NN Chernikova TM Suchkov YG Rudnev MM. Q fever and psittacosis in wild birds. Vopr Virusol. (1960) 5:586–91. 13687613

[105] Hubálek Z. Pathogenic microorganisms associated with gulls and terns (Laridae). J Vertebr Biol. (2021) 70:21009.1-98. 10.25225/jvb.2100930891612

[106] Vapniarsky N Barr BC Murphy B. Systemic coxiella -like infection with myocarditis and hepatitis in an eclectus parrot (*Eclectus roratus*). Vet Pathol. (2012) 49:717–22. 10.1177/0300985811409251 21712515

[107] Sander WE King R Graser W Kapfer JM Engel AI Adamovicz L. *Coxiella burnetii* in 3 species of turtles in the upper midwest, United States. Emerg Infect Dis. (2021) 27:3199–202. 10.3201/eid2712.211278 34808095 PMC8632166

[108] Yadav MP Sethi MS. Poikilotherms as reservoirs of Q-fever (*Coxiella burnetii*) in Uttar Pradesh. J Wildl Dis. (1979) 15:15–7. 10.7589/0090-3558-15.1.15 459042

[109] Pioz M Loison A Gauthier D Gibert P Jullien J-M Artois M. Diseases and reproductive success in a wild mammal: example in the alpine chamois. Oecologia. (2008) 155:691–704. 10.1007/s00442-007-0942-5 18189146

[110] Marreros N Hüssy D Albini S Frey CF Abril C Vogt H-R. Epizootiologic investigations of selected abortive agents in free-ranging alpine ibex (*Capra ibex ibex*) in Switzerland. J Wildl Dis. (2011) 47:530–43. 10.7589/0090-3558-47.3.530 21719818

[111] DeForge JR Cone LA. The serologic prevalence of Q fever (Coxiella *burnetii*) complement-fixing antibodies in the Peninsular bighorn sheep of Southern California. Am J Trop Med Hyg. (2006) 75:315–7. 10.4269/ajtmh.2006.75.315 16896140

[112] García E Espeso G Fernández R Gómez-Martín Á Rodríguez-Linde JM de la Fe C. *Coxiella burnetii* detected in three species of endangered North African gazelles that recently aborted. Theriogenology. (2017) 88:131–3. 10.1016/j.theriogenology.2016.09.019 27771116

[113] Lloyd C Stidworthy MF Wernery U. *Coxiella burnetii* abortion in captive dama gazelle (*Gazella dama*) in the United Arab Emirates. J Zoo Wildl Med. (2010) 41:83–9. 10.1638/2009-0005.1 20722258

[114] Fernández-Aguilar X Cabezón Ó Colom-Cadena A Lavín S López-Olvera JR. Serological survey of *Coxiella burnetii* at the wildlife–livestock interface in the Eastern Pyrenees, Spain. Acta Vet Scand. (2015) 58:26. 10.1186/s13028-016-0209-4 27121001 PMC4848809

[115] Hubálek Z Juticová Z Svobodová Š Halouzka J. A serologic survey for some bacterial and viral zoonoses in game animals in the Czech Republic. J Wildl Dis. (1993) 29:604–7. 10.7589/0090-3558-29.4.604 8258864

[116] Psaroulaki A Chochlakis D Angelakis E Ioannou I Tselentis Y. *Coxiella burnetii* in wildlife and ticks in an endemic area. Trans R Soc Trop Med Hyg. (2014) 108:625–31. 10.1093/trstmh/tru134 25163752

[117] Ioannou I Sandalakis V Kassinis N Chochlakis D Papadopoulos B Loukaides F. Tick-borne bacteria in mouflons and their ectoparasites in Cyprus. J Wildl Dis. (2011) 47:300–6. 10.7589/0090-3558-47.2.300 21441182

[118] Afema JA Beckmen KB Arthur SM Huntington KB Mazet JAK. Disease complexity in a declining alaskan muksox (*Ovibos moschatus*) population. J Wildl Dis. (2017) 53:311–29. 10.7589/2016-02-035 28099077

[119] Clemente L Fernandes TL Barahona MJ Bernardino R Botelho A. Confirmation by PCR of *Coxiella burnetii* infection in animals at a zoo in Lisbon, Portugal. Vet Rec. (2008) 163:221. 10.1136/vr.163.7.221 18708658

[120] Orynbayev MB Beauvais W Sansyzbay AR Rystaeva RA Sultankulova KT Kerimbaev AA. Seroprevalence of infectious diseases in saiga antelope (*Saiga tatarica tatarica*) in Kazakhstan 2012–2014. Prev Vet Med. (2016) 127:100–4. 10.1016/j.prevetmed.2016.03.016 27094147

[121] Candela MG Caballol A Atance PM. Wide exposure to *Coxiella burnetii* in ruminant and feline species living in a natural environment: zoonoses in a human–livestock–wildlife interface. Epidemiol Infect. (2017) 145:478–81. 10.1017/S0950268816002454 27776577 PMC9507642

[122] Yin MY Tan QD Qin SY Hu LY Liu GH Zhou DH. First serologic survey of Q fever in free- range yaks in China. Iran J Vet Res. (2015) 16:210–2. 27175178 PMC4827688

[123] Chomel BB Carniciu ML Kasten RW Castelli PM Work TM Jessup DA. Antibody prevalence of eight ruminant infectious diseases in California mule and black-tailed deer (*Odocoileus hemionus*). J Wildl Dis. (1994) 30:51–9. 10.7589/0090-3558-30.1.51 8151824

[124] Giovannini A Cancellotti FM Turilli C Randi E. Serological investigation for some bacterial and viral pathogens in fallow deer (*Cervus dama*) and wild boar (*Sus scrofa*) of the San Rossore preserve, Tuscany, Italy. J Wildl Dis. (1988) 24:127–32. 10.7589/0090-3558-24.1.127 2832622

[125] Smetanova K Schwarzova K Kocianova E. Detection of *Anaplasma phagocytophilum, Coxiella burnetii, Rickettsia spp*, and *Borrelia burgdorferi* s l. in ticks, and wild-living animals in Western and Middle Slovakia. Ann N Y Acad Sci. (2006) 1078:312–5. 10.1196/annals.1374.058 17114728

[126] González-Barrio D Fernández-de-Mera IG Ortiz JA Queirós J Ruiz-Fons F. Long-term dynamics of *Coxiella burnetii* in farmed red deer (*Cervus elaphus*). Front Vet Sci. (2015) 2:74. 10.3389/fvets.2015.00074 26697437 PMC4676194

[127] Baradel JM Barrat J Blancou J Boutin JM Chastel C Dannacher G. Bilan d'une enquête sérologique effectuée sur différents mammifères sauvages de France. Revue Scientifique et Technique de l'OIE. (1988) 7:861–83. 10.20506/rst.7.4.375 32370370

[128] Ebani VV Rocchigiani G Bertelloni F Nardoni S Leoni A Nicoloso S. Molecular survey on the presence of zoonotic arthropod-borne pathogens in wild red deer (*Cervus elaphus*). Comp Immunol Microbiol Infect Dis. (2016) 47:77–80. 10.1016/j.cimid.2016.06.003 27477510

[129] Kreizinger Z Szeredi L Bacsadi Á Nemes C Sugár L Varga T. Occurrence of *Coxiella burnetii* and Chlamydiales species in abortions of domestic ruminants and in wild ruminants in Hungary, Central Europe. J Vet Diagn Invest. (2015) 27:206–10. 10.1177/1040638714563566 25776545

[130] Tavernier P Sys SU de Clercq K de Leeuw I Caij AB de Baere M. Serologic screening for 13 infectious agents in roe deer (*Capreolus capreolus*) in Flanders. Infect Ecol Epidemiol. (2015) 5:29862. 10.3402/iee.v5.29862 26609692 PMC4660936

[131] Candela MG Serrano E Sevila J León L Caro MR Verheyden H. Pathogens of zoonotic and biological importance in roe deer (*Capreolus capreolus*): Seroprevalence in an agro-system population in France. Res Vet Sci. (2014) 96:254–9. 10.1016/j.rvsc.2014.02.003 24576494

[132] Blancou J. Serologic testing of wild roe deer (*Capreolus capreolus*) from the trois fontaines forest region of Eastern France. J Wildl Dis. (1983) 19:271–3. 10.7589/0090-3558-19.3.271 6358537

[133] Rijks JM Roest HIJ van Tulden PW Kik MJL Gröne A. *Coxiella burnetii* infection in roe deer during Q fever epidemic, the Netherlands. Emerg Infect Dis. (2011) 17:2369–71. 10.3201/eid1712.110580 22172398 PMC3311195

[134] Ejercito CLA Cai L Htwe KK Taki M Inoshima Y Kondo T. Serological evidence of *Coxiella burnetii* infection in wild animals in Japan. J Wildl Dis. (1993) 29:481–4. 10.7589/0090-3558-29.3.481 8355353

[135] Yates L Embil J Marrie TJ. Seroepidemiology of *Coxiella burnetii* among wildlife in nova scotia. Am J Trop Med Hyg. (1993) 49:613–5. 10.4269/ajtmh.1993.49.613 8250101

[136] Kirchgessner MS Dubovi EJ Whipps CM. Seroepidemiology of *Coxiella burnetii* in wild white-tailed deer (*Odocoileus virginianus*) in New York, United States. Vector Borne Zoonotic Dis. (2012) 12:942–7. 10.1089/vbz.2011.0952 22989183

[137] Jado I Carranza-Rodríguez C Barandika JF Toledo Á García-Amil C Serrano B. Molecular method for the characterization of *Coxiella burnetii* from clinical and environmental samples: variability of genotypes in Spain. BMC Microbiol. (2012) 12:91. 10.1186/1471-2180-12-91 22656068 PMC3413600

[138] Rozental T Ferreira MS Guterres A Mares-Guia MA Teixeira BR Gonçalves J. Zoonotic pathogens in Atlantic Forest wild rodents in Brazil: *Bartonella* and *Coxiella* infections. Acta Trop. (2017) 168:64–73. 10.1016/j.actatropica.2017.01.003 28077317

[139] Liu L Manxia H Ming L Chao J Yingqun F Yu Y. *Coxiella burnetii* in rodents on Heixiazi island at the Sino-Russian border. Am J Trop Med Hyg. (2013) 88:770–3. 10.4269/ajtmh.12-0580 23382172 PMC3617867

[140] Reusken C van der Plaats R Opsteegh M de Bruin A Swart A. *Coxiella burnetii* (Q fever) in *Rattus norvegicus* and *Rattus rattus* at livestock farms and urban locations in the Netherlands; could *Rattus* spp. represent reservoirs for (re)introduction? Prev Vet Med. (2011) 101:124–30. 10.1016/j.prevetmed.2011.05.003 21640416

[141] Runge M von Keyserlingk M Braune S Becker D Plenge-Bönig A Freise JF. Distribution of rodenticide resistance and zoonotic pathogens in Norway rats in Lower Saxony and Hamburg, Germany. Pest Manag Sci. (2013) 69:403–8. 10.1002/ps.3369 22888034

[142] Pascucci I di Domenico M Dall'Acqua F Sozio G Cammà C. Detection of lyme disease and Q fever agents in wild rodents in central Italy. Vector Borne Zoonotic Dis. (2015) 15:404–11. 10.1089/vbz.2015.1807 26134933 PMC4507354

[143] Thompson M Mykytczuk N Gooderham K Schulte-Hostedde A. Prevalence of the bacterium *Coxiella burnetii* in wild rodents from a Canadian natural environment park. Zoonoses Public Health. (2012) 59:553–60. 10.1111/j.1863-2378.2012.01493.x 22639912

[144] McQuiston JH Childs JE. Q fever in humans and animals in the United States. Vector Borne Zoonotic Dis. (2002) 2:179–91. 10.1089/15303660260613747 12737547

[145] Millán J Proboste T Fernández de Mera IG Chirife AD de la Fuente J Altet L. Molecular detection of vector-borne pathogens in wild and domestic carnivores and their ticks at the human–wildlife interface. Ticks Tick Borne Dis. (2016) 7:284–90. 10.1016/j.ttbdis.2015.11.003 26643497

[146] Gong X-Q Xiao X Liu J-W Han H-J Qin X-R Lei S-C. Occurrence and genotyping of *Coxiella burnetii* in Hedgehogs in China. Vector Borne Zoonotic Dis. (2020) 20:580–5. 10.1089/vbz.2019.2589 32301684

[147] Balti G Galon C Derghal M Souguir H Guerbouj S Rhim A. *Atelerix algirus*, the North African Hedgehog: suitable wild host for infected ticks and fleas and reservoir of vector-borne pathogens in Tunisia. Pathogens. (2021) 10:953. 10.3390/pathogens10080953 34451417 PMC8399139

[148] Widmer CE Azevedo FCC Almeida AP Ferreira F Labruna MB. Tick-borne bacteria in free-living jaguars (*Panthera onca*) in Pantanal, Brazil. Vector Borne Zoonotic Dis. (2011) 11:1001–5. 10.1089/vbz.2011.0619 21612532

[149] Potter AS Banazis MJ Yang R Reid SA Fenwick SG. Prevalence of *Coxiella burnetii* in Western Grey Kangaroos (*Macropus fuliginosus*) in Western Australia. J Wildl Dis. (2011) 47:821–8. 10.7589/0090-3558-47.4.821 22102652

[150] Duncan C Gill V Worman K Burek-Huntington K Pabilonia K Johnson S. *Coxiella burnetii* exposure in northern sea otters Enhydra lutris kenyoni. Dis Aquat Organ. (2015) 114:83–7. 10.3354/dao02857 25958809

[151] Minor C Kersh GJ Gelatt T Kondas A Pabilonia KL Weller CB. *Coxiella burnetii* in Northern fur seals and steller sea lions of alaska. J Wildl Dis. (2013) 49:441–6. 10.7589/2012-09-226 23568925 PMC6506168

[152] Duncan C Kersh GJ Spraker T Patyk KA Fitzpatrick KA Massung RF. *Coxiella burnetii* in Northern Fur Seal (*Callorhinus ursinus*) Placentas from St. Paul Island, Alask. Vector Borne Zoonotic Dis. (2012) 12:192–5. 10.1089/vbz.2011.0715 22017469

[153] Kersh GJ Lambourn DM Raverty SA Fitzpatrick KA Self JS Akmajian AM. *Coxiella burnetii* infection of marine mammals in the Pacific Northwest, 1997-2010. J Wildl Dis. (2012) 48:201–6. 10.7589/0090-3558-48.1.201 22247392 PMC11288310

[154] Shivaprasad HL Cadenas MB Diab SS Nordhausen R Bradway D Crespo R. *Coxiella*-like infection in psittacines and a toucan. Avian Dis. (2008) 52:426–32. 10.1637/8192-120707-Reg 18939630

[155] Stephen S Rao KN. Coxiellosis in reptiles of South Kanara district, Karnataka. Indian J Med Res. (1979) 70:937–41. 541017

[156] Paştiu AI Matei IA Mihalca AD D'Amico G Dumitrache MO Kalmár Z. Zoonotic pathogens associated with *Hyalomma aegyptium* in endangered tortoises: evidence for host-switching behaviour in ticks? Parasit Vectors. (2012) 5:301. 10.1186/1756-3305-5-301 23273169 PMC3564739

[157] Mendoza-Roldan JA Mendoza-Roldan MA Otranto D. Reptile vector-borne diseases of zoonotic concern. Int J Parasitol Parasites Wildl. (2021) 15:132–42. 10.1016/j.ijppaw.2021.04.007 34026483 PMC8121771

[158] Tokarz R Markotić A Margaletić J Lipkin WI Habuš J Jain K. Molecular survey of zoonotic agents in rodents and other small mammals in Croatia. Am J Trop Med Hyg. (2016) 94:466–73. 10.4269/ajtmh.15-0517 26711522 PMC4751941

[159] Aitken ID. Clinical aspects and prevention of Q fever in animals. Eur J Epidemiol. (1989) 5:420–4. 10.1007/BF00140132 2691272

[160] Foronda P Plata-Luis J del Castillo-Figueruelo B Fernández-Álvarez Á Martín-Alonso A. Serological survey of antibodies to *Toxoplasma gondii* and *Coxiella burnetii* in rodents in north-western African islands (Canary Islands and Cape Verde). Onderstepoort J Vet Res. (2015) 82:e1–4. 10.4102/ojvr.v82i1.899 26244685 PMC6238698

[161] Kersh GJ Lambourn DM Self JS Akmajian AM Stanton JB Baszler T. *Coxiella burnetii* infection of a steller sea lion (*Eumetopias jubatus*) found in Washington state. J Clin Microbiol. (2010) 48:3428–31. 10.1128/JCM.00758-10 20592144 PMC2937693

[162] Lapointe J-M Gulland FM Haines DM Barr BC Duignan PJ. Placentitis due to *Coxiella burnetii* in a pacific harbor seal (*Phoca vitulina richardsi*). J Vet Diagn Invest. (1999) 11:541–3. 10.1177/104063879901100612 12968740

[163] OIE. Manual of Diagnostic Tests and Vaccines for Terrestrial Animals. Chapter 3.1.17. Q Fever. Paris: OIE (2018). Available online at: https://www.woah.org/fileadmin/Home/eng/Health_standards/tahm/3.01.17_Q_FEVER.pdf

[164] Guatteo R Seegers H Taurel A-F Joly A Beaudeau F. Prevalence of *Coxiella burnetii* infection in domestic ruminants: a critical review. Vet Microbiol. (2011) 149:1–16. 10.1016/j.vetmic.2010.10.007 21115308

[165] Arricau-Bouvery N Souriau A Bodier C Dufour P Rousset E Rodolakis A. Effect of vaccination with phase I and phase II *Coxiella burnetii* vaccines in pregnant goats. Vaccine. (2005) 23:4392–402. 10.1016/j.vaccine.2005.04.010 16005747

[166] Hogerwerf L van den Brom R Roest HIJ Bouma A Vellema P Pieterse M. Reduction of *Coxiella burnetii* prevalence by vaccination of goats and sheep, the Netherlands. Emerg Infect Dis. (2011) 17:379–86. 10.3201/eid1703.101157 21392427 PMC3166012

[167] Long CM. Q fever vaccine development: current strategies and future considerations. Pathogens. (2021) 10:1223. 10.3390/pathogens10101223 34684172 PMC8539696

[168] Berri M Rousset E Champion JL Russo P Rodolakis A. Goats may experience reproductive failures and shed *Coxiella burnetii* at two successive parturitions after a Q fever infection. Res Vet Sci. (2007) 83:47–52. 10.1016/j.rvsc.2006.11.001 17187835

